# Identifying opportunities for upstream evaluations relevant to child and maternal health: a UK policy-mapping review

**DOI:** 10.1136/archdischild-2022-325219

**Published:** 2023-03-31

**Authors:** Emma Stewart, Anna Pearce, Joanne Given, Ruth Gilbert, Sinead Brophy, Richard Cookson, Pia Hardelid, Katie L Harron, Alastair Leyland, Rachael Wood, Ruth Dundas

**Affiliations:** 1 MRC/CSO Social and Public Health Sciences Unit, University of Glasgow, Glasgow, UK; 2 School of Nursing and Paramedic Science, Ulster University, Coleraine, Londonderry, UK; 3 Population, Policy and Practice Department, UCL Great Ormond Street Institute of Child Health, London, UK; 4 School of Medicine, Swansea University, Swansea, UK; 5 Centre for Health Economics, University of York, York, North Yorkshire, UK; 6 Public Health Scotland, Edinburgh, UK; 7 Usher Institute, The University of Edinburgh Usher Institute of Population Health Sciences and Informatics, Edinburgh, UK

**Keywords:** child health, child development

## Abstract

**Objective:**

Interventions to tackle the social determinants of health can improve outcomes during pregnancy and early childhood, leading to better health across the life course. Variation in content, timing and implementation of policies across the 4 UK nations allows for evaluation. We conducted a policy-mapping review (1981–2021) to identify relevant UK early years policies across the social determinants of health framework, and determine suitable candidates for evaluation using administrative data.

**Methods:**

We used open keyword and category searches of UK and devolved Government websites, and hand searched policy reviews. Policies were rated and included using five criteria: (1) Potential for policy to affect maternal and child health outcomes; (2) Implementation variation across the UK; (3) Population reach and expected effect size; (4) Ability to identify exposed/eligible group in administrative data; (5) Potential to affect health inequalities. An expert consensus workshop determined a final shortlist.

**Results:**

336 policies and 306 strategy documents were identified. Policies were mainly excluded due to criteria 2–4, leaving 88. The consensus workshop identified three policy areas as suitable candidates for natural experiment evaluation using administrative data: pregnancy grants, early years education and childcare, and Universal Credit.

**Conclusion:**

Our comprehensive policy review identifies valuable opportunities to evaluate sociostructural impacts on mother and child outcomes. However, many potentially impactful policies were excluded. This may lead to the inverse evidence law, where there is least evidence for policies believed to be most effective. This could be ameliorated by better access to administrative data, staged implementation of future policies or alternative evaluation methods.

**WHAT IS ALREADY KNOWN ON THIS TOPIC**

- In the UK, a plethora of policies and strategies target child and maternal health, with a demonstrable political will to invest in the early years.
- The social determinants of health framework identifies ‘upstream’ or sociostructural factors such as childcare, schools, housing, welfare, transport, health and social care, and the workplace as important elements affecting child health and health inequalities.
- Political devolution in the UK has led to policy divergence. This offers an opportunity to conduct natural experiment evaluations that determine the impact of upstream policies on child health and health inequalities.

**WHAT THIS STUDY ADDS**

- This comprehensive review of UK early years policies across the social determinants of health domain contributes to tackling the inverse evidence law in maternal and child health research (where the least evidence is available for settings where need is greatest).
- The review identifies three policy priority areas in child and maternal health for future research: welfare grants in pregnancy and early childhood, early years education and childcare, and Universal Credit and welfare policies.
- Many child policies were found not to be suitable for natural experimental evaluation, which could lead to the inverse evidence law.

**HOW THIS STUDY MIGHT AFFECT RESEARCH, PRACTICE OR POLICY**

- There is a need for better access to administrative data (eg, on eligibility criteria) and staged implementation of future policies (affording greater cross-country variation).
- This study offers a framework to distil and prioritise candidate policies for natural experiment methods using administrative data.

## Introduction

Giving every child the best start in life is a key policy goal for a healthy society. Government strategies and policies identify childhood and pregnancy as crucial stages for policy intervention. Commitment to children’s rights and well-being is evident across the historical strategies of the four UK nations: *Every Child Matters* 1 (England and Wales), *Getting it Right for Every Child* 2 (Scotland) and *Children and Young People’s Strategy* 3 4 (Northern Ireland). Recently, attention on the early years has gathered momentum with emphasis on the *First 1000 days of life*,^5^ referring to the crucial period from conception to a child’s second birthday that is optimal for intervening to improve child health. As part of the UK Government’s levelling up policy agenda, the Leadsom Early Years Healthy Development Review (2020) set out recommendations in *The Best Start for Life: A Vision for the 1001 Critical Days* 6 and the proposed family hubs are being rolled out in England.^7^

### Social determinants of child health

The social determinants of children’s health are key to improving health outcomes. The social determinants of health (SDoH) framework identifies the inter-relationship between parents, communities and the wider macro level context.^8^ So in addition to health policies, such as health visiting programmes, ‘upstream’^9^ sociostructural policy interventions are key to tackling the root causes of poor child health. At the structural level, living and working conditions influence children’s health either directly, or indirectly through their parents. This includes childcare, schools, housing, welfare, transport, health and social care, and the workplace. Existing research evidence documents the impact of housing,^10^ income^11^ and welfare reform^12^ on child and maternal health. Policy interventions focusing on ‘upstream’ factors, such as poverty and welfare, can significantly improve child and maternal health outcomes.^13–16^ Nevertheless, there tends to be stronger evidence about individual-level clinical interventions than population-level social interventions with larger population health impacts.^17^ This ‘inverse evidence law’, whereby the availability of good evidence tends to vary inversely with the need for it in the population served, also applies to early childhood interventions.

### Evaluating policies in the early years

All four UK nations are politically and financially committed to early years interventions. Yet a divergence in philosophical stance combined with devolved decision making has led to deviation in policies and priorities.^18^ This presents an opportunity to explore how diverse policy landscapes potentially impact on child health outcomes. Early years policies cannot always be evaluated in trial settings so natural experimental designs offer a strong alternative. Policy variations can be evaluated within the relatively homogeneous context of the UK, namely the wider political framework (UK central government) and health service environment (the UK National Health Service). Administrative data sets can be exploited for these evaluations as they cover the whole population and have a long time series. For example, previous research projects have used natural experiment methodologies and administrative data to evaluate the impact of policies such as the Health in Pregnancy Grant,^19 20^ the Healthy Start Voucher Scheme^21^ and the effect of lone parent obligations.^22^ Research has also exploited UK variations in staged policy implementation to evaluate policies.^23–25^

To contribute towards tackling the inverse evidence law in maternal and child health, we conducted a comprehensive review of the UK policy landscape to identify candidate early years policies for possible future evaluation. We endeavoured to identify all government (not just child health focused) policies that potentially affect maternal and child health across the SDoH framework; and determine suitable candidates for evaluation using administrative data. There is great possibility for natural experimental evaluations based on policy variation in content, timing and implementation across the four UK nations, including but not limited to those enabled by political devolution.

## Method

During Spring/Summer 2020, we mapped the policy landscape in the four UK nations to identify national variations that potentially impact on child and maternal health outcomes. Both national and devolved policy owners were identified. This included the UK policy landscape (covering England and Wales) as well as the devolved administrations in Scotland, Wales and Northern Ireland. The policy topic areas were aligned with the SDoH framework: welfare, employment, health, housing, education and the environment. This was supplemented with a search of the four Public Health Agencies in 2021.

### Identification: search strategy

Two search methods were adopted for each national government website: (1) Open keyword search: ‘child’, ‘child health’, ‘child and maternal health’ and (2) Category search: categories selected based on the SDoH (listed above) with all subcategories inspected. Key department websites relevant to the SDoH were included, for example, Departments of Health, Housing, Education, Environment and Social Security across all UK nations. The category search of department websites covered policies, development plans, frameworks and legislation. The Public Health Agencies in the UK nations were similarly searched using category and open keyword searches.

When using the open keyword search, sites typically reported numerical results. Some sites, for example, Scottish Government and NI Direct, were searched manually due to multiple links being listed on each separate web page. In these instances, cases are reported as the total number of web pages searched. Each website was manually searched until saturation point was reached, that is, no new policies were identified. The search strategy was iterative with the identification of policies in one nation prompting an equivalent search in the other UK nations.

The search strategy was supplemented with hand searching of existing policy horizon scanning documents and multiple reports published by the British Academy Childhood Policy Programme. Additionally, existing public health policy reviews and evaluations of interventions were examined to identify any other relevant policies.

### Screening: exclusion/inclusion criteria

The life course periods in the early years can be divided into pregnancy, infancy and preschool. Any policy that could impact on maternal or child health during these early years periods was included. Early years policies were identified from the 1940s until 2021. Due to data availability, we excluded all policies before 1981 and limited the search of earlier decades. To direct the search strategy, inclusion and exclusion criteria were further defined (see online supplemental table 1). Data were extracted for each policy and recorded in Excel.

10.1136/archdischild-2022-325219.supp1 Supplementary data

### Filtering: essential criteria

The Maternal and Child Health Network (MatCHNet) is a multidisciplinary community of public health researchers, methodologists, policy makers and service providers. The network aims to harness cross-country administrative data to evaluate national policy impacts on maternal, infant and child health, and health inequalities across the four UK nations. MatCHNet is one of four networks funded by the UK Prevention Research Partnership. During several workshops, MatCHNet’s expert group determined essential criteria to form the basis for subsequent filtering. The aim was to identify (include) whole national policies that could potentially be evaluated using administrative data. We discussed necessary elements to conduct policy evaluations and derived five essential criteria: (1) Potential for policy to affect maternal and child health outcomes; (2) Implementation variation across the UK; (3) Population reach and expected effect size; (4) Ability to identify exposed/eligible group in administrative data (while time is a key factor to identifying populations exposed to certain policies, there is also the need to identify eligible populations, for example, lone parents for lone parent obligations); and (5) Potential to affect health inequalities. The group determined the key filtering criteria were 2–4, with criteria 1 and 5 used to prioritise policies for analysis after the initial filtering.

All policies were reviewed and assigned essential criteria by ES, and subsequently reviewed by AP, JG and RG. Policies were defined as being included or excluded as potential candidates for future evaluation. The filtering focused on essential criteria 2 (whole nation policies that vary across the UK) as well as criteria 3 and 4 (proportion of population to benefit/effect size and identifying the exposed/eligible group in administrative data). This process was based on judgement and consensus was agreed for each policy by at least two of the above reviewers. The input of an additional reviewer was sought where a decision could not be made.

### Prioritisation: shortlisting policies for future evaluation

The policy prioritisation exercise was based on discussions at three stakeholder consultations (held during 2021), an online prioritisation poll (51 responses) and a final consensus workshop. The stakeholder group consisted of representatives from central and local government, public health agencies, service providers, and third sector organisations from across the four UK nations. Online poll respondents were 39% academics, 35% National Health Service, with representation from government departments (4%), the voluntary sector (12%) and service provision (2%). At the final consensus workshop, our expert group identified a shortlist of policies for potential future evaluation, which included consideration of the remaining criteria 1 (potential to impact on child and maternal health) and 5 (potential to impact on health inequalities). The expert group also considered previous and ongoing policy evaluations to ascertain knowledge gaps and policy areas requiring further investigation.

## Results

### Screening and filtering

**Figure 1:**
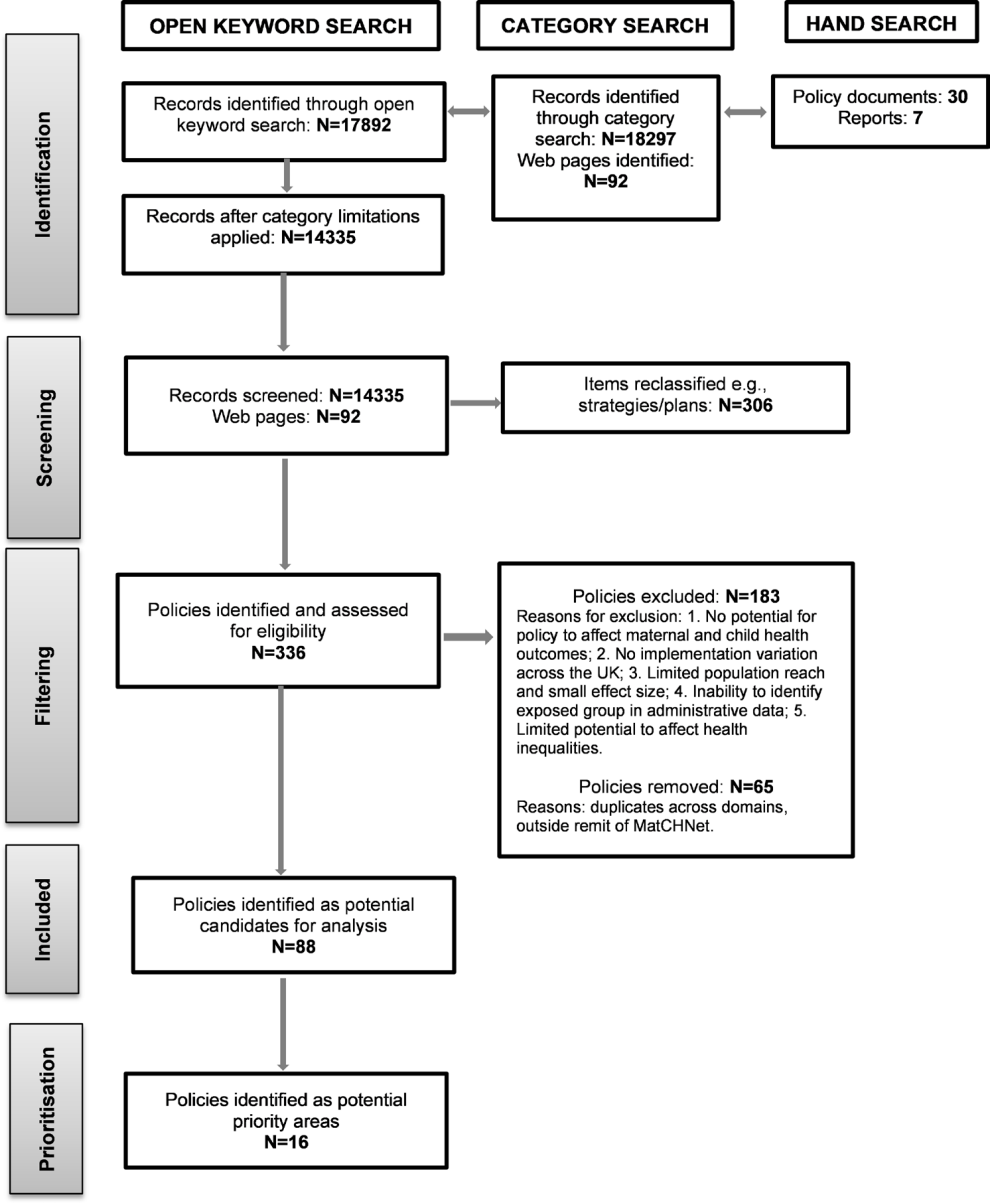
Flow diagram summarising identification, screening, filtering, and prioritisation of UK policies relevant to child and maternal health. MatCHNet, Maternal and Child Health Network.

The search strategy identified 17 892 records from open keyword searches and 18 297 from the category search (figure 1). After category limitations were applied (eg, publications, research, policy papers), 14 335 records were eligible for screening along with 92 web pages. The screening also included hand searching of 30 policy documents and 7 British Academy reports.^26–32^ The search process identified 306 items that were excluded and reclassified as strategies, action plans or legislation. After the initial screening process, a total of 336 policies were eligible for further assessment.

**Figure 2:**
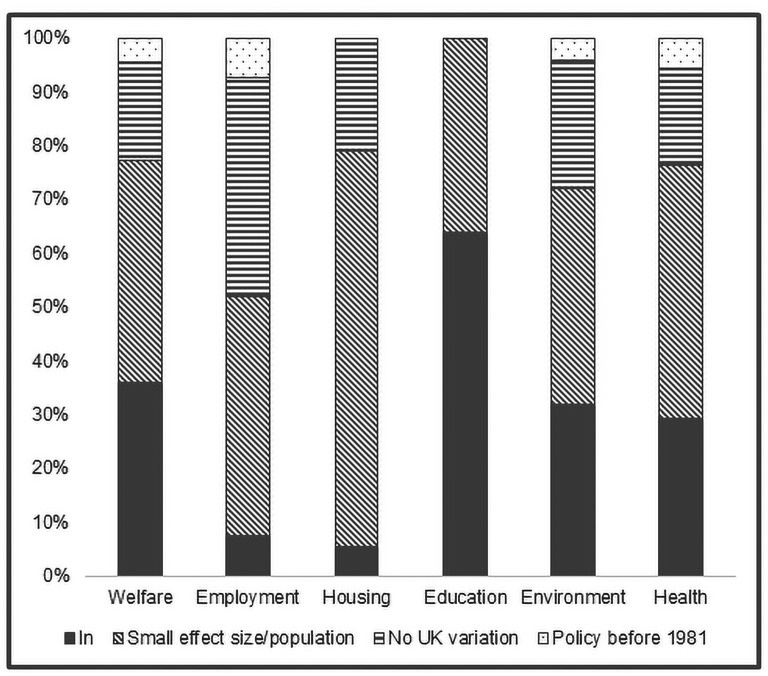
Policy filtering decisions by social determinants of health domains (n=271). MatCHNet, Maternal and Child Health Network

After filtering (criteria 2–4), 88 policies were found to vary across the four UK nations (across the six domains). During the filtering process, 65 records were removed. These were either duplicates across the domains (eg, some housing policies were reclassified as welfare policies) or met the exclusion criteria on closer inspection. Examining the filtering decisions across the social determinants domains indicates heterogeneity in terms of suitability for future analysis (see figure 2). Some 64% of education policies were included with 36% excluded due to small effect size and population reach. By contrast, polices included in the other domains are significantly less, 36% welfare, 32% environment, 29% health, 7% employment and only 5% of housing policies. The main reasons for excluding policies across these domains were small effect size and population reach.

### Identifying policy priorities

**Figure 3:**
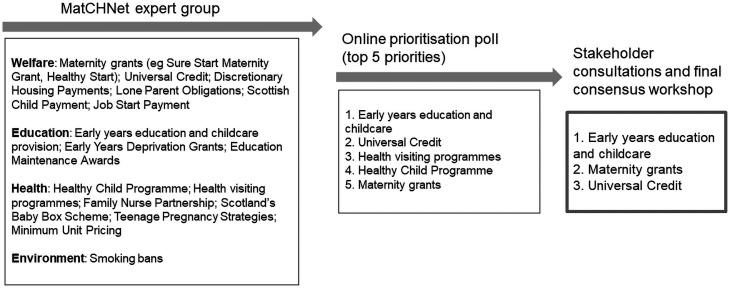
Shortlisted candidate policies by social determinants of health domains (n=16). MatCHNet, Maternal and Child Health Network.

The 16 shortlisted candidate policies (figure 3) were in the domains of welfare, education, health and the environment. Our online prioritisation poll (Summer 2021) identified five key policies, which formed the basis for discussion at the three stakeholder consultations. Being mindful of population reach and expected effect size (essential criteria 3), the expert group selected priority policy areas rather than individual interventions. Based on the essential criteria and input from MatCHNet’s stakeholders, the final consensus workshop identified three policy areas as suitable candidates for natural experimental evaluation using administrative data (see table 1): Pregnancy grants (welfare), Early years education and childcare (education) and Universal Credit (welfare). Detailed explanation of how these policy areas vary across the four UK nations are reported elsewhere.^33–35^

**Table 1:** Summary description of early years policy priority areas identified as suitable candidates for natural experimental evaluation*

1. Welfare grants in pregnancy and early childhood	
*Sure Start Maternity Grant (2000) (England, Wales, and Northern Ireland) (2000–2018, Scotland*)	One-off welfare payment of £500 given to low-income pregnant women to help with costs of having a child. From 2012, limited to the first child.
*Best Start Grant (2018) (Scotland*)	Three payments for low-income pregnant women and families. *Pregnancy and Baby Payment*: £642.35 for first child and £321.20 for each subsequent child. *Early Learning Payment*: £267.65 for the parent/carer of a child who is between 2 years and 3½ years old. *School Age Payment*: £267.65 given to help with costs of a child entering school.
*Healthy Start (2006) (England, Wales, and Northern Ireland) (2006–2019, Scotland*)	Means-tested weekly vouchers given to pregnant women, children under 1 year and children aged 1–3 years to buy fruit, vegetables and milk. Means-tested pregnancy vitamins.
*Best Start Foods (2019) (Scotland*)	Means-tested weekly vouchers given to pregnant women, children under 1 year and children aged 1–2 years to buy fruit, vegetables and milk. Universal pregnancy vitamins.
2. Early years education and childcare provision	
*Universal entitlement*	All four UK nations offer a universal entitlement for 38 weeks per year, ranging from 10 hours to 30 hours per week, for children aged 3–4 years.
*Working entitlement*	Working families are entitled to 30 hours per week in England (2017) and Wales (2019).
*Disadvantaged entitlement*	All four UK nations offer extra provision for disadvantaged 2-year-old children. Hours range from 7.5 hours to 30 hours per week. Disadvantaged provision is part of wider services in Northern Ireland (Sure Start) and Wales (Flying Start).
*Targeted early years funding*	Extra support given to early years providers to support disadvantaged children aged 3–4 years in England (*Early Years Pupil Premium* £302 per child), Wales (*Early Years Pupil Development Grant* £1150 per child) and Northern Ireland (*Pathway Fund* up to £15 000 or £15 000–30 000 per provider).
3.Universal Credit and welfare policies	
*Universal Credit (2013*)	Combines six different welfare payments. Roll-out began in England and Wales (2013), followed by Scotland (2015) and Northern Ireland (2017).
*Household Benefit Cap (2013*)	Cap limits amount of welfare payments. Set at £20 000 per year for couples and single parents since 2016.
*Removal of Spare Room Subsidy (2013*)	Housing benefit reduced if rented property is judged to have more bedrooms than necessary (also referred to as ‘bedroom tax’).
*Two Child Limit (2017*)	Withdraws means-tested support from third and subsequent children born since April 2017.
*Scottish Child Payment (2021*)	£10 weekly payment to tackle child poverty for families with children under 6 years (means tested). Increased to £20 per week from April 2022. From November 2022, increased to £25 per week and extended to all children under 16 years in low-income families.

*More detailed information on how these policies vary across the four UK nations in terms of implementation, timing and policy eligibility is available in MatCHNet’s policy briefings and reports.^33–35^

MatCHNet, Maternal and Child Health Network.

The three policy areas are broadly similar across countries but differ in timing of implementation and exposed populations, offering opportunities for evaluation of effectiveness (see table 2). For example, welfare grants in pregnancy and early childhood have recently changed in Scotland with the introduction of the Best Start Grant (2018) and Best Start Foods (2019). Early years education and childcare varies across the four UK nations by hours of provision and target populations (ranging from universal to low income/working families). Finally, the four UK nations have implemented diverse mitigation measures in response to the roll-out of Universal Credit that may have different effects on the health of mothers and children.

**Table 2:** Filtering criteria of early years policy areas identified as suitable candidates for natural experimental evaluation

*Essential filtering criteria*	*1. Welfare grants in pregnancy* *and early childhood*	*2. Early years education* *and childcare*	*3. Universal Credit* *and welfare policies*
*1. Expected health outcomes*	Birth outcomes; child physical/cognitive development; child diseases—diabetes, obesity; child mental health; maternal mental health	Child physical/cognitive development; child diseases—obesity; child mental health; maternal mental health	Birth outcomes; child diseases—diabetes, obesity; child mental health; maternal mental health
*2. Variation across UK*	Variation in Scotland since 2018	Provision varies across four UK nations	Mitigation measures and differential effects
*3. Population reach and effect size*	Medium	Large	Large
*4. Exposed/eligible group*	Low-income pregnant women; Parents/children up to aged 5 years (low-income families)	Preschool children (aged 2–4 years); low income/working families	Benefit recipients
*5. Affect health inequalities?*	Yes	Yes	Yes

## Discussion

From our extensive searching, we narrowed 336 policies and 306 strategy documents to 88 policies across the SDoH domains. Most included policies were in the welfare domain and other prominent domains were education, environment and health. After consultation and consensus workshops, we identified three policy areas as suitable candidates for natural experimental evaluation using administrative data: pregnancy and early childhood grants (welfare), early years childcare (education) and Universal Credit (welfare).

### Limitations of child and maternal health policy making

A closer inspection of the excluded policies illustrates key challenges for policy evaluation using administrative data. First, many policies are focused on local and community interventions, which leads to local variances in service provision.^36^ Variabilities in provision and exposure make it difficult to undertake cross-country comparisons. Examples include: The Dundee Families Project,^37^ a local housing community intervention that aimed to help homeless families; and the Opportunity Area Programme,^38^ covering 12 areas of England.

Second, the small proportion of the population exposed to policies leads to challenges in conducting data analysis; for example, welfare policies supporting full-time students or single parents with childcare costs (Childcare Grant (England, Northern Ireland and Wales); Lone Parents’ Childcare Grant (Scotland)). While these grants may have a significant effect on individual families and the respective groups may be identifiable from birth registrations and education data, the small populations are challenging for data analysis. Likewise, homelessness prevention policies are problematic to evaluate since homeless people are difficult to identify from administrative data or not included in some data sources.

Finally, associated with the above issue is the complication of determining the exact interventions that are funded. The Child and Families Delivery Grant (Wales)^39^ is undoubtedly important for child and maternal health but there are difficulties in identifying eligible groups as well as understanding the degree of change from previous grant funding. These examples highlight the practical difficulties in determining how a policy is/was implemented and operationalised at the local level as a basis for cross-national comparison and evaluation using administrative data.

### Limitation of search strategy

In the absence of a definitive guide, we conducted a comprehensive overview of policies around child and maternal health in the UK. The search strategy, nonetheless, does have limitations. While the filtering process was based on expert judgement, there is limited evidence for some of the policy effects. The uniformity of search across the websites was hampered by platform heterogeneity, requiring adaptability and iterative hand searching. The dynamic nature of the platforms also creates challenges to replicating the search strategy.

Searching policy sites in this manner potentially omits past policy developments, with websites focused on current policies. Additional reports and policy reviews are necessary to supplement historical details. Reliance on policy titles can potentially exclude relevant interventions, for example, the Northern Ireland Extended Schools Programme^40^ includes awards for preschool settings. Finally, as with all reviews, the search parameters are time-limited. A policy-mapping database requires continual updating to reflect changes or developments.

## Conclusion

Political devolution and a relatively homogeneous health and services policy environment in the UK presents a unique opportunity for child and maternal health researchers. A wide-ranging search of the UK policy landscape identified valuable opportunities to evaluate upstream, sociostructural impacts on mother and child outcomes. Three key policy priority areas in child and maternal health were identified for future evaluation.

However, this process highlighted many potentially impactful policies that did not meet the criteria for natural experimental evaluation, which could lead to the inverse evidence law. This could be ameliorated by better access to administrative data (eg, on eligibility criteria), staged implementation of future policies (affording greater cross-country variation) or alternative evaluation methods (eg, simulations).

## Data Availability

Data are available upon reasonable request. For access to the extracted data, requests should be submitted to the corresponding author for consideration. Access may be granted following review.
