# Supplementary material for: Identifying opportunities for upstream evaluations relevant to child and maternal health: a UK policy-mapping review
Source: Arch Dis Child. 2023 Mar 31;108(7):556–62. doi: 10.1136/archdischild-2022-325219 (PMC10314013; doi:10.1136/archdischild-2022-325219)
Supplement: Supplementary data [file archdischild-2022-325219supp001.pdf]

Supplementary Table 1: Description of search strategy, screening criteria and extraction

**Research question**	Identification and mapping of UK and national policies that potentially impact upon child and maternal health outcomes
**Data sources**	Policy owners (UK Government, Irish Assembly, Scottish Government, Welsh Assembly) AND relevant Departments<br>Existing public health policy reviews (Horizon scanning by Jenny Woodman/CPRU)<br>The Childhood Policy Programme (The British Academy)<br>Evaluations of policy interventions
**Search criteria**	**Government websites**<br>1. *Open keyword search*<br>'Child', 'child health', 'child and maternal health'<br><br>2. *Category search (Executive level)*<br>(categories selected based upon social determinants of health)<br>*UK/England* (www.gov.uk): filtering based on categories: Childcare and Parenting (Pregnancy and Birth, Financial Help, Childcare, Schools and Education)<br><br>*Ireland* (www.nidirect.gov.uk): Benefits and Money, Environment and the Outdoors, Employment, Training and Careers, Health and Well-Being, Family, Home and Community, Property and Housing, Travel, Transport and Roads, Education (www.northernireland.gov.uk): Children and Young People, Community and Society, Employment, Equality, Rights & Citizenship, Health, Housing, Public Health, Regeneration, Schools, Transport, Welfare<br><br>*Scotland* (www.gov.scot): Children and Families, Economy, Education, Equality and Rights, Environment and Climate Change, Health and Social Care, Housing, Transport<br><br>Wales (www.gov.wales): Children and Families, Communities and Regeneration, Education and Skills, Environment and Climate Change, Equality, Health and Social Care, Housing, Transport<br><br>*3. Publication search (Department level):* Departments of Education, Health, Economy, Environment, Social Security Scotland etc.<br><br>**Public Health Agencies** in all 4 UK nations: open keyword (as above) and category searches of publications.<br><br>**Existing literature/reports**<br>Public health policy reviews on child and maternal health in 4 UK nations<br>Intervention evaluations of child and maternal health policies in 4 UK nations
**Inclusion criteria**	*Geographical coverage*: UK, England, Northern Ireland, Scotland, and Wales<br>*Early years periods*: pregnancy, infancy, pre-school<br>*Year of policy implementation*: from 1981 to present, 1995-2021 (before/after New Labour in 1997)<br>*Policy area (SDH)*: welfare, employment, health, housing, education, and environment<br>*Population coverage*: national level policies<br>*Type of policy*: fiscal, regulation or education, communication, and information<br>*Funding*: UK or National government
**Exclusion criteria**	*Geographical coverage*: policies outside UK<br>*Early years periods*: policies aimed directly/solely at children older than 6 years of age<br>*Year of policy implementation*: before 1981 (due to data availability)

	Policy area: dental policies<br>*Population coverage*: local/city level policies, minority groups (e.g., asylum seekers/travellers)<br>*Type of policy*: National reports (e.g., One Health), action plans, policy strategies<br>*Funding*: non-government funded programmes e.g., National Lottery Funding
**Data extracted**	Policy name, Policy area, Time period, Geographical coverage, Year of policy, Type of policy (fiscal, regulation or education, communication and information), Short descriptor, URL, (Author for Policy Documents)

Stewart E, *et al. Arch Dis Child* 2023; 108:520–526. doi: 10.1136/archdischild-2022-325219
